# Multi-Target Potential of Berberine as an Antineoplastic and Antimetastatic Agent: A Special Focus on Lung Cancer Treatment

**DOI:** 10.3390/cells11213433

**Published:** 2022-10-31

**Authors:** Ijeoma Theresa Achi, Paromita Sarbadhikary, Blassan P. George, Heidi Abrahamse

**Affiliations:** Laser Research Centre, Faculty of Health Sciences, University of Johannesburg, P.O. Box 17011, Johannesburg 2028, South Africa

**Keywords:** apoptosis, autophagy, berberine, lung cancer, phytochemicals, signaling pathways, cell proliferation, metastasis

## Abstract

Despite therapeutic advancements, lung cancer remains the principal cause of cancer mortality in a global scenario. The increased incidence of tumor reoccurrence and progression and the highly metastatic nature of lung cancer are of great concern and hence require the investigation of novel therapies and/or medications. Naturally occurring compounds from plants serve as important resources for novel drugs for cancer therapy. Amongst these phytochemicals, Berberine, an alkaloid, has been extensively explored as a potential natural anticancer therapeutic agent. Several studies have shown the effectiveness of Berberine in inhibiting cancer growth and progression mediated via several different mechanisms, which include cell cycle arrest, inducing cell death by apoptosis and autophagy, inhibiting cell proliferation and invasion, as well as regulating the expression of microRNA, telomerase activity, and the tumor microenvironment, which usually varies for different cancer types. In this review, we aim to provide a better understanding of molecular insights of Berberine and its various derivative-induced antiproliferative and antimetastatic effects against lung cancer. In conclusion, the Berberine imparts its anticancer efficacy against lung cancers via modulation of several signaling pathways involved in cancer cell viability and proliferation, as well as migration, invasion, and metastasis.

## 1. Introduction

Cancer is one of the most life-threatening disorders reported globally as being the disease with the highest death rate after cardiovascular diseases [1,2]. Globally, the number of patients with cancer most likely will increase significantly in the next 50 years as a result of certain demographic influences/changes such as population ageing and growth. The new cases and deaths due to cancer were estimated at 19.3 and 10.0 million according to a GLOBOCAN 2020 update, two- to three-fold more in developed countries compared to developing countries for both males and females [3]. Although, by incidence rate, lung cancer ranks second after breast cancer, it is still the leading cause of cancer-related mortality globally, with an estimated 1.8 million mortalities (18%). The global cancer burden is expected to rise to 28.4 million cases in 2040, which is a 47% increase from 2020, with a marked increase in developing countries (64% to 95%) as compared to developed countries (32% to 56%) as a result of demographic changes; however, this may worsen in the future as a result of socio-economic and demographic transitions [3].

**Figure 1:**
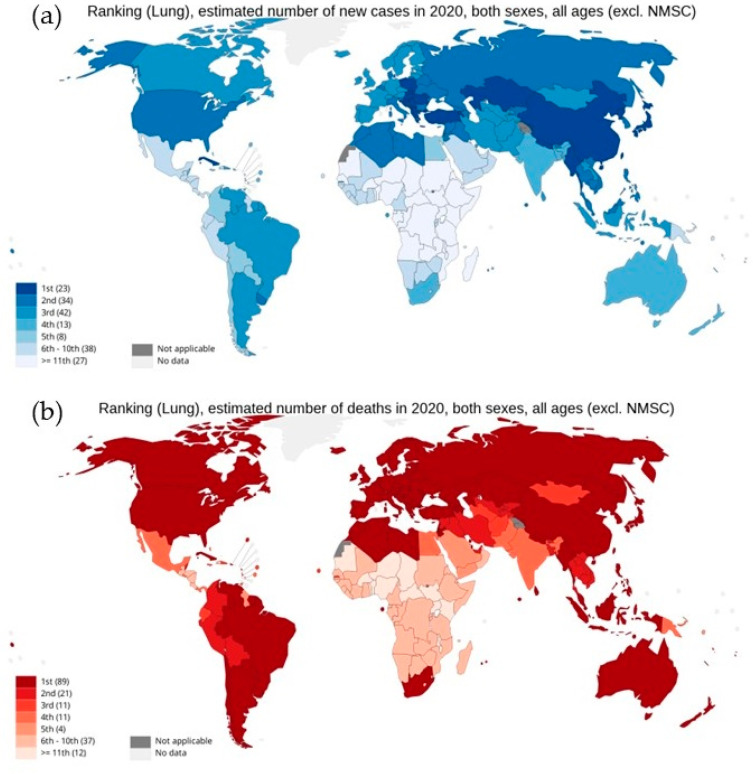
National ranking for lung cancer: (**a**) estimated new cases in 2020, (**b**) estimated number of deaths in 2020, in both sexes as per GLOBOCAN cancer statistics 2020. Graph production: IARC (http://gco.iarc.fr/ 18 August 2022), World Health Organization.

As per the GLOBOCAN report 2020, with an estimated 2.3 million new cases globally, lung cancer remains the second most commonly diagnosed cancer, with an ~11.4% incidence rate in all cancer types in both sexes. However, as shown in Figure 1, lung cancer represents the principal cause of cancer-associated deaths globally, with the highest mortality rate of 18% [3,4,5,6,7]. Lung cancer arises in many different sites in the bronchial tree, and depending on its anatomic location, symptoms and signs of lung cancers are highly variable. Almost 70% of lung cancer patients are diagnosed with advanced stage disease (stage III or IV). Therefore, the treatment becomes a challenge, with a five-year survival rate of 19.4%, which is far lower than many leading cancer types, such as prostate (98.8%), breast (89.8%), and colorectal (64.4%) [5,8]. Moreover, high levels of histological and cellular heterogeneity in lung cancer play an important role in the development of therapeutic resistance [9]. Histologically, lung cancer is categorized into two different forms: non-small cell lung cancer (NSCLC) and small cell lung cancer (SCLC). About 85% of lung cancers are NSCLC, which is any type of epithelial lung cancer and is further classified based on the type of cell found in the tumor, generally into three subtypes: adenocarcinoma (ADC), squamous cell lung carcinoma (SqCC), and large-cell (undifferentiated) lung carcinoma (LCLC) [7]. Although the survival rate for five years for NSCLC from detection is 24%, for patients with metastatic NSCLC the rate drops to only 6% [10]. While SCLC is the most aggressive, high-grade neuroendocrine carcinoma represents about 15% of all cases of lung cancers [11].

The major approaches employed in the treatment of lung cancer include surgery, radiotherapy, chemotherapy, and endocrine therapy [8,12]. Surgery is the primary treatment option for resectable early-stage lung cancer patients with small tumors without lymph node involvement and offers 5-year survival rates of 60–80% for stage I NSCLC and 30–50% for stage II NSCLC patients [8]. However, for highly aggressive SCLC, the outcome of surgery remains controversial, even for limited stage disease, as studies have shown almost 23–25% of patients report pathological upstaging from clinical stage I to stage II or III following surgical resection [13]. Similarly, postoperative radiotherapy is not usually recommended for completely resected tumors or for stage I and II patients due to its induced serious cardiopulmonary toxicity with poorer survival rates [7]. For NSCLC patients, benefit of postoperative adjuvant platinum drug-based chemotherapy varies from a 3% decrease in the risk of death in high-risk stage IB patients to a 13% increase in benefits for stage IIIA disease, still with a higher risk of metastatic disease relapse. However, the adjuvant radiation therapy is shown to be harmful for NSCLC stage IB and II disease due to increased radiation-induced mortality risk and is less likely to provide modest benefit in stage IIIA [14,15]. Similarly, a combination of chemoradiation for inoperable limited stage SCLC and platinum-based chemotherapy followed by thoracic and cranial irradiation in extensive stage SCLC also suffers from relapse-refractory progression. Although, at these stages, the standard first-line etoposide and cisplatin or carboplatin offers response rates of about 70%, the progression-free survival remains very poor, at only 5.5 months [16]. However, targeted and non-targeted cytotoxic chemotherapy against stage IV metastatic lung cancer suffers from limitations. For example, platinum-based chemotherapy alone results in modest improvements in patients’ survival. Similarly, clinical studies showed no significant differences in overall survival with platinum-based two-drug chemotherapy regimens with paclitaxel and gemcitabine. Among the targeted therapy, oral tyrosine kinase inhibitors (TKI) with promising therapeutic outcome and improved disease-free survival rates show evidence of primary and acquired resistance in most patients over time. Treatment with cetuximab, an epidermal growth factor receptor inhibitor, in combination with platinum-based chemotherapy can only be offered to specific NSCLC patients with *EGFR*-positive tumors, which also increases the risk of febrile neutropenia. Similarly, bevacizumab (an angiogenesis inhibitor) therapy is restricted to certain subpopulations of NSCLC patients, with several associated adverse effects [17]. Thus, the shortcomings of standard lung cancer treatments with respect to their efficacy, safety, and resistance emphasize the need for improved and novel agents and/or treatment strategies [18].

Among several different potent and safer anticancer phytochemicals, Berberine represents a promising therapeutic option against lung cancer. Herein, we present a review from available published investigations to provide an in-depth understanding of the molecular mechanism of the anticancer and antimetastatic potential of Berberine as a prospective drug candidate for the treatment of lung cancer.

## 2. Phytocompounds: Boon for Lung Cancer Therapy

For many decades, natural phytoproducts have been be the bedrock of traditional medicinal approaches and the main components of the lead molecules in drug discovery due to their curative and preventive health benefits [19]. Natural compounds have gained considerable attention as preventive and therapeutic agents against cancer because many of the bioactive phytocompounds have been reported to have antineoplastic efficacies, and some of them have been approved for clinical use [18,20,21,22,23]. A crucial part of their activity is the tendency to regulate several survival pathways with fewer toxic side effects [24]. Worldwide challenges to recent treatment of lung cancer are springing up due to acquired resistance, which has become the major cause of mortality for lung cancer patients [25,26]. Furthermore, it is noted that several plant crude extracts or isolated phytochemicals, such as piperines, flavonoids, and tannins, act as chemosensitizers or resistance regulators [27]. Despite the advanced research, the use of synthetic drugs against resistant lung cancer is limited [28,29].

**Figure 2:**
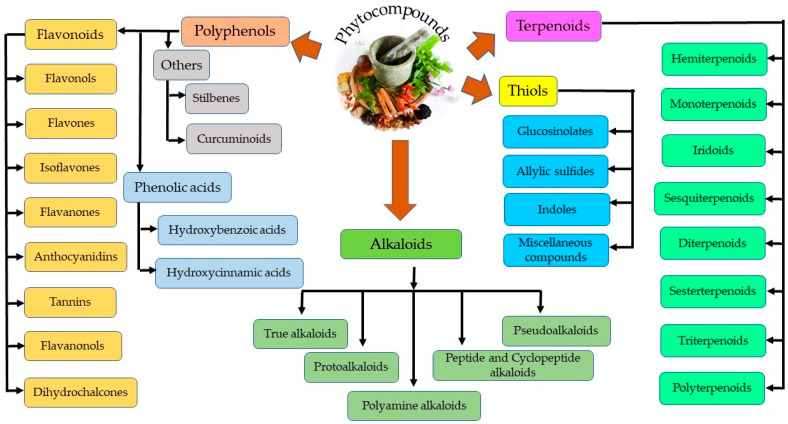
Different classes of phytocompounds.

Plant secondary metabolites can be categorized into four distinct groups—thiols, alkaloids, polyphenols, and terpenoids, as represented in Figure 2 [30]. Polyphenols are further grouped into phenolic acids and flavonoids. Flavonoids are the most abundant and common polyphenols, having a wide spectrum of pharmacological importance. Phenolics are the most regular and diverse plant chemicals in terms of structure. The hydroxyl (-OH) group contained by this plant chemical usually bonds with the aromatic hydrocarbon groups. Phenolics are generally known for their antioxidant contributions towards guiding against free radical–linked diseases in humans [31]. Tannins seem difficult to describe chemically, since they cuts across a series of polymers and oligomers; however, they belong to the group of heterogenous polyphenolic compounds of remarkable molecular weight, having the tendency of forming complexes with proteins, alkaloids, minerals, polysaccharides, and nucleic acids reversibly or irreversibly [32]. Terpenoids, another natural compound, derives from 5-carbon isoprene units. Members of this group differ from one another either by the fundamental carbon skeleton or functional groups. They are nearly ubiquitous (found almost in all the classes of living things) and hence are the largest occurring group of natural secondary metabolites [33]. On the other hand, alkaloids, being highly bioactive, can also cause detrimental effects in the cases of higher doses. For example, vinca alkaloids have been utilized in a series of cancer treatments, including lung, via cell death induction at the metaphase as well as binding to microtubulins, especially β-tubulin, thereby preventing polymerization and microtubule assemblage. Vinorelbine and vindesine have been approved clinically for their effectiveness in the remedy of many oncological issues, such as breast and lung cancer, Kaposi’s sarcoma, advanced testicular cancers, Hodgkin and non-Hodgkin’s lymphomas, and leukemia, mostly in synergism with other chemotherapeutics [34]. Thiols are relatively less distributed in plants compared to other phytochemicals (alkaloids and terpenes) [35].

Numerous plant extracts and phytochemicals or their analogues have been used to inhibit the proliferation of cancer cells by inhibiting the rate of cell proliferation and initiating cell death by targeting numerous vital signaling pathways, affecting glucose homeostasis and controlling cell growth. This offers potential as a promising anticancer agent in controlling cancer cell growth and progression. Clinically used plant-derived anticancer compounds usually belong to four major classes: vinca alkaloids, taxane diterpenoids, camptothecin derivatives, and epipodophyllotoxin [21,36]. Moreover, out of 247 antitumor drugs approved until 2019, around 21%, 7.3%. 0.4% and 17.4% are respectively biological macromolecules, unaltered natural products, natural product botanicals, and natural product derivatives, which shows the great potential of anticancer phytochemicals in future cancer research and treatment [37].

Phytochemicals like Berberine, Quercetin, Lycopene, Sulforaphane, Curcumin, Resveratrol, and Epigallocatechin gallate are currently in clinical trials. While other phytochemicals under pre-clinical studies include 6-Shogaol, Allicin, Alpinumisoflavone, Andrographolide, Apigenin, Baicalein, Baicalin, Curcumin, Decursin, Decursinol, Dicumarol, Epigallocatechin, Emodin, Genistein, Gingerol, Glycyrrhizin, Hispidulin, Licochalcone A, Nimbolide, Physapubescin B, Pterostilbene, Resveratrol, Sulforaphane, Thymol, Thymoquinone, Ursolic acid, Withaferin A, etc. [21,30,38]. Most importantly, few have been approved for the treatment of lung cancer, including Camptothecins (topoisomerase I inhibitors), Taxanes and Vinca alkaloids (Tubulin inhibitors), and Podophyllotoxins (Tubulin/Topoisomerase II inhibitors) [30,39].

## 3. Berberine

Among the numerous secondary metabolites of plant, alkaloids seem to have pharmacological properties of varying degrees [40]. One of the tremendously explored ancient phytomedicines/phytochemicals is Berberine, an isoquinoline alkaloid present in the crude extracts and decoctions of bark, roots, and stems of several plant species; it has been extensively studied as a bioactive phytochemical with anticancer potential [41]. Several researchers have reported the effectiveness of Berberine against arrays of ailments: hypertension, diabetes, metabolic syndrome, obesity, diarrhea, fatty liver and coronary artery diseases, gastroenteritis, hyperlipidemia, Alzheimer’s disease, and cancer [42,43,44]. 

### 3.1. Chemistry

**Figure 3:**
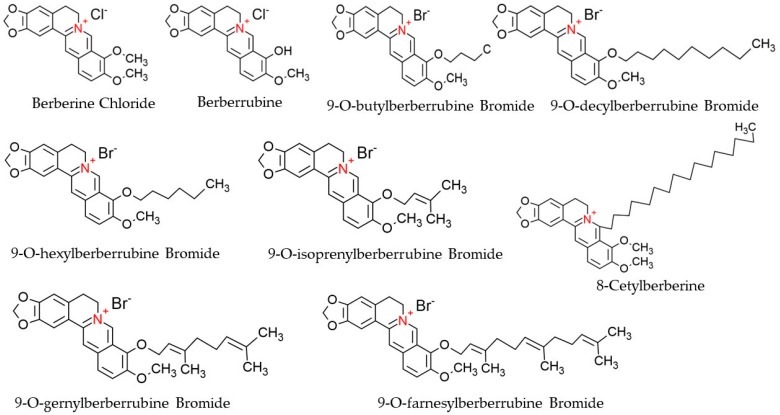
Chemical structures of Berberine and its derivatives.

Berberine (C_20_H_18_NO_4_), a derivative of 5,6-dihydro-dibenzo[a,g] quinolizinium, is a quaternary ammonium salt of an isoquinoline alkaloid origin and is regarded as a crucial bioactive component of many plant species, such as Chinese schneid, Berberis aristata, and Coptis japonica [45]. It has a higher solubility in hot water compared to ethanol or cold water but has zero solubility in chloroform, ether, benzene, and all other solvents of organic origin [46]. Structurally, Berberine is comprised of a dihydroisoquinoline ring and isoquinoline ring, as shown in Figure 3. According to Leyva-Peralta et al., 2020, Berberine’s scaffold can be grouped into four rings, namely rings A, B, C, and D, where rings C2 and C3 of the A ring form a major group, the methylenedioxy group, which happens to be implicated in the majority of Berberine’s biological activities, including its antineoplastic potentials [47]. The quaternary ammonium structure contained in C ring, particularly the N+ in the aromatic ring, is imperative for its effect against bacterial infection [48]. At the moment, there is an increasing focus on the C and D rings of Berberine with regard to their structural modification [49,50]. Furthermore, alkylation of Berberine at positions C8 and C13 were described by Singh and colleagues (Singh et al., 2021). Again, N7 and C13 positions are susceptible to modifications, which eventually enhance the anticellular proliferative activities of Berberine [48]. Additionally, Berberine exhibits a maximum absorption wavelength of 350 nm and an emission wavelength of 530 nm [51].

### 3.2. Pharmacokinetics (Bioavailability and Metabolism) and Safety

The measurement of the bioavailability of any molecule is often calculated through its pharmacokinetic profile, which includes absorption, metabolism, biodistribution, and elimination (excretion). As Berberine is poorly absorbed and quickly eliminated from the body due to low bioavailability, high doses of Berberine need to be administered to achieve the therapeutic effects [52]. This reduction in bioavailability is further enhanced due to its binding with plasma proteins; eventually, only the fraction of unbounded Berberine is left to reach the target tissues [53,54,55,56]. Previously, it was shown that Berberine has an absolute bioavailability of 0.37% when given in a single dose via an oral route in rats of varying body weights (48.2, 120, or 240 mg/kg) [57]. Similarly, following the study of Sahibzada and colleagues, it was shown that the Cmax (mean maximum plasma concentration) in rabbit after a single oral dose of 50 mg/kg of Berberine was 0.411 µg/mL [58]. Similarly, a low level of absorption of Berberine was also recorded for human beings, where an oral administration of ~400 mg of Berberine showed a Cmax value of 0.4 ng/mL after 8 h of administration [59]. Another study carried out on 10 healthy individuals showed a remarkably low level of Cmax of 0.07 Nano moles after oral administration of 500 mg of Berberine [60]. The low in vivo bioavailability of Berberine is mainly explained by its thorough elimination during the first pass in the intestine, where the drug is taken out of circulation by the hepatic tissue due to its quick metabolism in the hepatic organ and elimination via feces, hence leading to a decreased level of systemic circulation [61]. This metabolism often takes place through two main phases. Phase 1 metabolism, which is achieved by cytochrome CYP450 in the liver, usually leads to the production of four metabolites: berberrubine (M1), thalifendine (M2), demethyleneBerberine (M3), and andjatrorrhizine (M4) [62]. The demethylation of Berberine to its metabolites M1 and M2 is achieved through the enzymes CYP3A1/2 and CYP2AB, respectively. The coalescence of phase 1 metabolites with sulfuric acid is often enhanced by enzymes such as transferases, which leads to the production of phase 2 (thalifendine) metabolites. Biodistribution studies of Berberine revealed the presence of a very low concentration of Berberine metabolites in the blood stream, whereas higher levels were present in the hepatic organ (Liver), kidneys, muscles, brain, pancreas, lungs, and heart, with very minimal distribution in the adipose tissues and with an excretion rate of 22.83%, 22.74%, and 0.0939% in the bile, feces, and urine, respectively. [53,54,55,56,63,64,65,66].

In general, Berberine usage does not lead to side effects such as cytotoxic, genotoxic and mutagenic activities. However, it can lead to complications in the gastrointestinal tract in a concentration-dependent manner [67]. In addition, the findings of Jiang et al. and Caliceti et al. showed that Berberine’s binding to macrolides can lead to arrhythmias [68,69]. Berberine is not recommended during pregnancy or for infants owing to its substitution ability, whereby it substitutes bilirubin for albumin [70]. Furthermore, it was reported that Berberine’s metabolism by hepatic enzymes is easier at low doses as compared to higher doses, which can reduce the functionality of the cytochrome P450 [71].

## 4. Potential Therapeutic Effects of Berberine on Lung Cancer

Berberine offers various pharmacological activities, such as anti-inflammatory, antioxidant, anti-lipidemic, anti-diabetic, and pro-apoptotic activities, because of its ability in regulating cellular signaling pathways, cell cycle arrest, and DNA interaction [72]. The in vivo and in vitro anticancer effects on NSCLC are via apoptosis induction, proliferation prevention, arresting cell cycle, and control against tumorigenesis [73,74]. Similarly, Berberine derivatives such as 9-0-alkyl--,13-alkyl, and 13-0-alkyl Berberine bromide exhibited antineoplastic ability against different human cancer cell lines by inhibiting cell proliferation [10,75,76,77,78]. In addition, Berberine derivatives such as Berberine (B1), Berberrubin (B2), and 9—position substituted berberrubine derivatives (B3 to B7) demonstrated a strong dose- and time-dependent growth inhibition activity against NSCLC, A549, H23, and H1435 lung cancer cells post treatment [10]. Table 1 represents the therapeutic potency and induced anticancer mechanism of reported Berberine derivatives in lung cancer cell lines. 

**Figure 4:**
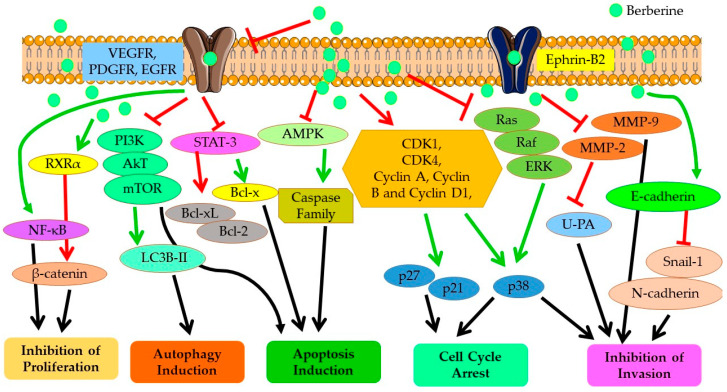
Anticancer mechanisms and signaling pathways regulated by Berberine. Red line shows inhibition/reduction, green line shows promotion/induction.

As represented in Figure 4, Berberine imparts its anticarcinogenic effect by preventing cancer cell proliferation, inducing cell cycle arrest at different phases, and downregulating Cyclin and Kinase proteins. Berberine contributes to both programmed cell death (apoptosis) and autophagy in cancer cells by upregulating the expressions of caspase proteins and other pro-apoptotic pathways and proteins, and targeting AMPK/mTOR/ULK1 pathways. It modulates the expression of many genes partaking in tumorigenesis and inflammation, including nuclear factor-Kappa B (*NF-KB*), *cyclin D1*, B-Cell lymphoma 2 (*Bcl-2*), intercellular adhesion molecule-1 (*ICAM-1*), tumor necrosis factor (*TNF*), cyclooxygenase *(COX)-2* and DNA topoisomerase I and II [41,80]. Berberine also inhibits cancer cell invasion and metastasis by affecting several different signaling pathways to restrain the expression of MMP-2 and MMP-9 proteins. Further, Berberine alters the tumor microenvironment by affecting various inflammatory responses and immune molecules. Other than its direct anticarcinogenic potential, Berberine imparts anti-inflammatory and antioxidant effects, contributing to the overall outcome of its anticancer effectiveness. Recent studies have shown that the inhibitory potential of Berberine also involves interaction with miRNAs involved in tumorigenesis (cancer cell proliferation, invasion, and metastasis) and inhibits telomerase activity [81]. Thus, overall, Berberine imparts a strong suppressive effect on NSCLC proliferation, growth, and metastasis through diverse mechanisms, which are discussed in detail in the following sections. 

### 4.1. Molecular Targets for Antineoplastic Potential

Numerous researchers are currently working to reveal the molecular mechanisms of Berberine’s activity; most findings pinpoint the ability of Berberine to tightly bind to deoxyribonucleic acid (DNA), whereas others suggest nuclear and cytoplasmic targets [82]. Berberine has direct interaction with nucleic acids and several proteins and/or genes, such as NF–κb, p53, telomerase, MMPs, estrogen receptors, and DNA topoisomerase I. Generally, treatment with Berberine leads to cell cycle arrest and subsequent mortality in several human neoplastic cell lines, as well as marked expression of apoptotic factors. However, the mechanisms of action through which Berberine exerts its effects seem ambiguous; upon administration, Berberine tends to suppress the activation of different proteins and/or modulate the expression of many genes partaking in tumorigenesis and inflammation [83,84]. Similarly, Berberine alters lipid and glucose metabolism [85]. Mechanistic insights into the antiproliferative and antimetastatic properties in several in vitro cell lines and in vivo lung cancer models are summarized in Table 2.

#### 4.1.1. Cell Cycle Arrest and Apoptosis

Berberine has been shown to induce cell cycle arrest and/or cell death via apoptosis through altering the expression pattern of several genes and signaling pathways involved in the regulation of cell cycle progression and apoptosis [50]. In one study, the antiproliferative and cytotoxic potential of Berberine against NSCLC A549 cells was exhibited through activation of the p38α MAPK signaling pathway, with subsequent induction in tumor suppressor *p53* and transcription factor Forkhead homeobox type O3a (FOXO3a) protein expressions. Further, the activation of FOXO3a, a transcription factor with tumor suppressor activity, followed by an increase in expression of cyclin-dependent kinase inhibitor p21 (CIP1/WAF1) protein resulted in cell cycle arrest in G0/G1 along with inducing the apoptotic cell death pathway [96]. Fu et al. demonstrated that activation of caspase and PARP proteins along with the release of mitochondrial cytochrome c upon Berberine treatment leads to cytochrome c/caspase-dependent apoptosis in lung cancer cells [89]. Similarly, Berberine inhibited the growth of H460 lung cancer cells via the induction of G0/G1 cell cycle arrest and cell death [97]. Berberine derivatives 9-O-decylberberrubine bromide, 9-O-dodecylberberrubine bromide, 9-O-gernylberberrubine bromide, and 9-O-farnesylberberrubine bromide have been shown to exert inhibition of in vitro tumorigenesis in NSCLC cells via G0/G1 cell cycle arrest by induction of CDK2 and CDK4 and reduction of p21 expression. However, an anticancer effect was not induced by apoptosis or by partial apoptosis; rather, it was mediated by cellular autophagy [10,77].

In a comparative study, to understand the chemotoxic potential of Berberine on lung cancer cells with different p53 statuses, Berberine treatment was tested against two different human NSCLC cancer cell lines, A549 with wild-type p53 (p53+/+) and p53-deficient H1299 cells (p53−/−). The results showed that Berberine inhibited cell proliferation and induced apoptotic mediated cell death in both the cell lines; however, the magnitude of the response was somewhat lower in H1299 cells compared to A549 cells. Further, mechanistically, Berberine treatment upregulates the expression of pro-apoptotic Bax and Bak proteins and downregulates the antiapoptotic proteins Bcl-2 and Bcl-xl in both A549 and H1299 cells. Thus, the increase in the *Bax/Bcl-2* ratio causes an increase in cytochrome c and Smac/DIABLO release from mitochondria, which subsequently results in activation of caspase 3 to induce apoptotic cell death. Similarly, the inhibitory effect of Berberine administration was more pronounced in A549 tumor xenografts, where the tumor volume was reduced by 58% and 79% at a concentration of 100 and 200 mg/kg, respectively, as compared to H1299 xenograft tumors, where the tumor volume was reduced by 35% and 62% with same drug dose [74]. Oral dietary administration of Berberine resulted in p53-independent tumor growth arrest in an in vivo lung cancer model by inducing G1 cell-cycle arrest via inhibiting the proliferative kinase signaling axis comprising Akt, CREB, and MAPK [88]. Another study reported by Chem et al. showed the p53-independent prominent cell inhibitory effect of Berberine treatment on human NSCLC cell lines NCI-H460, A549 with wild type *p53* expression, and p53-deficient NCI-H1299 cells. The study showed that Berberine acts as a promising DNA damage inducer, resulting in significant apoptosis both in vitro and in vivo in human NSCLC models, whereby Berberine significantly accelerated the degradation of the SWI-independent-3 transcription regulator family member A (Sin3A) protein. This subsequently downregulates the mRNA and protein level of DNA Topoisomerase II beta (TOP2B) involved in inducing DNA double-stranded breaks, and accumulation of TOP II-mediated DNA damage contributes to cancer cell death [91].

Suppression of Berberine-mediated AP-1 transcriptional activity, inhibiting the expression of c-Jun, and the binding of transcription factors to the *CCND1* gene AP-1 motif has shown to downregulate the cyclin D1 expression, leading to cell cycle arrest in human pulmonary giant cell carcinoma cell line [98]. In addition, 8-cetylBerberine, a Berberine derivative with improved bioavailability and pharmacological properties, exhibited better antineoplastic potency compared to Berberine, with almost ~10 times lower IC_50_ concentration against A549 cells. However, similar to Berberine, 8-cetylBerberine prompted (i) significant apoptotic cell death by remarkably upregulating the cleavage of caspase 3, caspase 8, and caspase 9, suggesting the involvement of both intrinsic and extrinsic apoptotic pathways; (ii) G0/G1 phase cell cycle arrest by reducing the expression level of Cyclin D1 and Cyclin E1 proteins. Molecular mechanism results showed that 8-cetylBerberine treatment downregulated the intracellular levels of an active form of phosphorylated-Akt and PI3K, while increasing the protein expression of Cyclin-dependent kinase inhibitors *p21* and *p27*, suggesting the inhibitory effect of 8-cetylBerberine on the PI3K-Akt signal pathway to induce apoptosis and antiproliferation in lung cancer cells. Further, the studies on an in vivo A549 xenograft mouse model presented a very strong tumor growth reduction, with inhibition rates of 34.58% and 59.07% for 5 and 10 mg/kg administrated doses of 8-cetylBerberine, respectively, compared to only a 27.47% inhibition rate with a 120 mg/kg Berberine dose. As compared to Berberine, 8-cetylBerberine appeared to be a safer drug for in vivo administration, as evaluated by its negligible to normal long-term toxicity as well as absence of any significant differences in organ coefficients of lung, heart, liver, spleen, and kidney upon 8-cetylBerberine treatment compared to control untreated mice groups. Further, the findings in this report also suggest an important role of 8-cetylBerberine in interfering with the initiation and progression of lung cancer, assessed from three important lung cancer–associated serum biomarkers: neuron-specific enolase (NSE), cytokeratin-19 (CYFRA 21-1), and carbohydrate antigen 125 (CA125). The 8-cetylBerberine treatment decreased NSE, CYFRA 21-1, and CA125 levels to 37.7%, 24.4%, and 63.8% respectively, in serum samples of mice compared to tumor control [79]. Further, Berberine inhibited cell proliferation, reduced DNA synthesis, and induced apoptosis in NSCLC cell lines NCI-H460 and NCI-H1975 by markedly suppressing both phosphorylated and total levels of STAT3 protein to reduce the expression of downstream target gene cyclin D1, which is a crucial cell cycle regulator for the G1/S phase. Moreover, in this study, Berberine exhibited its potential to reduce Cancer Stem-like Cells (CSCs) in lung cancer cells by repressing the expression levels of CSC-related oncoprotein c-Myc downstream to the STAT3 pathway [99].

Berberine has been shown to induce apoptosis in NSCLC by inducing ROS augmentation or oxidative stress and subsequent activation of Apoptosis Signal-regulating Kinase 1 (ASK1)/JNK signaling as well as the mitochondrial apoptotic pathway [86]. ASK1, a member of the mitogen-activated protein kinase (MAPK) family, regulates cell differentiation and apoptosis [66]. Upon activation, ASK1 dissociates from thioredoxin-1 to activate c-jun-NH2-kinase (JNK) and p38 MAPK pathways, which further induces cell death [100]. Berberine-induced ROS generation promotes ASK1/JNK phosphorylation, which in turn suppresses Bcl-2 family proteins, driving a loss of MMP, the release of mitochondrial cytochrome c, and the consequent caspase-dependent apoptosis pathway [86]. A different mechanism of action of Berberine was elucidated by Chen et al., whereby Berberine induces cell apoptosis and suppresses NSCLC cell proliferation and growth both in vitro and in vivo models by modulating the miR-19a/TF/MAPK signaling pathway induced by Berberine-mediated upregulation of miR-19a and downregulation of tissue factor (TF) expression [90]. Zheng et al. further showed the Berberine-induced lung cancer cell proliferation inhibition and G2/M cell cycle arrest is mediated by reducing the expressions of transcription factor SP1 and 3-phosphoinositide-dependent protein kinase-1 (PDPK1), which was suggested to prevent the interaction between the two, resulting in the inhibition of a downstream effector DNA methyltransferase 1 (*DNMT1*) expression involved in tumor growth and progression [101].

For the very first time, Kalaiarasi et al. reported the ability of Berberine to induce sub-G0/G1 cell cycle arrest by epigenetic reprogramming in a lung cancer cell line, where it was shown to downregulate total histone deacetylases (HDACs) and modulate the activity of class I, II, and IV HDACs through histone hyperacetylation. Moreover, suppression of *COX-2* activation mediated by Berberine treatment was correlated with ROS generation, alteration in mitochondrial membrane potential (Δψm), and activation of apoptotic cell death. In addition, *Bax* activation induced by Berberine resulted in the alteration of Δψm, leading to p53-mediated cytochrome c release, subsequently triggering the activation of the caspase-mediated apoptotic pathway [73].

#### 4.1.2. Effect on DNA Repair

In a conflicting mechanism, a study reported by Ni et al. showed that apoptosis is not the only required mode of action for Berberine-induced cell proliferation inhibition in NSCLC cells. They demonstrated Berberine-mediated cell cycle arrest at the G1/S phase in human NSCLC cell lines, A549, H1299, and H1975 through downregulating DNA replication and repair pathways. Results showed suppression at both mRNA and protein level expressions of ribonucleotide reductase catalytic subunit M1 (*RRM1*) and ribonucleotide reductase regulatory subunit M2 (*RRM2*) of the ribonucleotide reductase enzyme, which plays a central role in the DNA synthesis pathway involved in generating purine and pyrimidine deoxynucleotide. With respect to DNA repair, suppression in expression levels of DNA ligase 1 (*LIG1*) and DNA polymerase epsilon 2 (*POLE2*), which encodes the B subunit of DNA polymerase ε, affected overall nuclear DNA replication and repair as well as the cell cycle process. Further, the in vivo studies with Lewis lung carcinoma (LLC) cells in a transplanted tumor mouse model showed that in comparison to the control untreated group, Berberine treatment resulted in significantly higher tumor necrosis alongside a reduction in the expression levels of *RRM1*, *RRM2*, *LIG1*, and *POLE2* [102]. In continuation of the study, Ni et al. analyzed the differential expression of genes in Berberine-treated A549 cells and in the lung adenocarcinoma cohort created by the Cancer Genome Atlas (TCGA) and suggested that Berberine interferes with tumor cell proliferation and progression primarily by downregulating the cell cycle and DNA replication related genes [92]. An overall suppression in the expression of mini-chromosome maintenance complex component 4 *(MCM4*), DNA polymerase alpha subunit 2 *(POLA2*), *POLE2*, and DNA primase subunit 1 (PRIM1) in A549 cells treated with Berberine, which are involved in G1/S cell cycle transition and DNA replication, was observed. Thus, as a mechanism, both in vitro and in vivo lung adenocarcinoma xenografts studies indicated Berberine-mediated downregulation of forkhead box protein M1 (FoxM1), an oncoprotein transcription factor, majorly inhibiting the *POLE2* expression involved in DNA replication and affecting the overall survival of lung adenocarcinoma [92].

#### 4.1.3. Influence on Telomerase

Other than the direct inhibitory effect of Berberine on molecular targets involved in cell cycle arrest and apoptosis-mediated cell death pathways, Berberine has been shown to exert its antineoplastic effect via inhibiting human telomerase reverse transcriptase (hTERT) signaling. Several studies have demonstrated that the poor survival and prognosis of lung cancer is highly associated with overexpression of hTERT, a catalytic subunit of the telomerase enzyme involved in the synthesis of telomeric DNA and maintaining chromosomal stability. The high activity and overexpression of hTERT results in cellular immortalization and malignant transformation and thus is regarded as the landmark of carcinogenesis [103,104]. Fu et al. showed that Berberine treatment in NSCLC cells suppressed the expression of activating enhancer-binding protein-2 (AP-2) factors AP-2a and AP-2b proteins, which subsequently diminished their binding to hTERT promoter and therefore downregulated the expression of tumor-related genes including *hTERT*, *PI3K*/*Akt*, and *Raf/MEK/ERK hTERT* [89].

#### 4.1.4. Other Anticancer Targets

Other than the well-recognized mechanisms discussed above, several other studies report the expanding targets of Berberine-mediated anticancer effects. Berberine has been shown to suppress Cyclooxygenase (COX)-2 signaling by repressing the expression of *COX-2* both at the protein and mRNA level and partially by preventing the binding of *NF-κB* to the *COX-2* promoter [89]. This inhibitory role of Berberine in overexpressing *COX-2* ultimately impacts multiple pathways involved in tumor growth and malignant progression, including angiogenesis, resistance to apoptosis, tumor invasiveness, and metastases, as well as host immunity mediated by the *COX-2*/prostaglandin (PGE) E2 signaling axis [105]. Further, Berberine also inhibits the Raf/MEK/ERK mitogen-activated protein kinase (MAPK) pathway and PI3K/Akt signaling pathway, most probably by preventing the phosphorylation of Akt and ERK proteins and downregulating HIF-1α signaling [89]. Abnormal activation of both the signal transduction pathways results in unregulated cancer cell proliferation and migration, inhibition of apoptosis, and therapeutic resistance and has been strongly linked with tumorigenesis, disease progression, and poor prognosis in lung cancer [106,107]. Fan et al. demonstrated the selective action of Berberine against Gefitinib-resistant NSCLC cell lines H1975 and H1650, by modulating the ROS/AMPK pathway to suppress sterol regulatory element-binding protein 1 (SREBP-1) and lipogenesis, thus inhibiting cancer cell proliferation [108].

Berberine treatment has shown to have importance not only in the treatment of cancer but also in preventing cancer initiation. A study by Xi et al. showed that Berberine suppresses the N-acetyltransferase *(NAT*) expression at transcriptional and protein levels as well as reduces its activity in the A549 cell line, suggestive of decreasing the probability of causing lung cancer upon exposure to chemical carcinogens. NAT is a xenobiotic enzyme that catalyzes biotransformation of an arylamine carcinogen 2-amino-fluorene (2-AF) into N-acetyl-2-aminofluorene (2-AAF). This 2-AAF is further metabolically activated by Cytochrome P-450 to N-hydroxy-acetylaminofluorene (N-OH-AAF). N-OH-AAF interacts with cellular DNA to form the covalent Arylamine–DNA adduct, inducing mutagenic and carcinogenic effects [109].

Other than inducing strong antiproliferative and cell killing effects at higher concentrations by apoptosis and inflammatory aggravation, Berberine treatment at lower concentrations revealed the probability of induction of quiescence in A549 cells, as indicated by the presence of a weak senescence-associated β-galactosidase (SA-β-gal) activity (a prominent senescence marker), higher expression of CDK inhibitor *p21WAF1*, a lack of senescence-associated secretory phenotype (SASP); i.e., absence of effective PI3K/Akt/mTOR pathway induction and low expression of cyclins are characteristics associated with quiescence. Thus, this study presented a very important outlook for understanding the difference between the overlapping nature of quiescence and senescence induced by lower doses of Berberine, which needs to be considered before implying pro-senescence attributes of Berberine in cancer therapeutics [110].

### 4.2. Molecular Targets for Antimetastatic Potential

One of the major factors for lung cancer, especially NSCLC, being the leading cause of all cancer deaths, is its fast progression potency and metastatic ability to spread readily in its early stages to a wide range of organs of different anatomy and physiology [111,112]. Metastasis is a complex multi-step process encompassing invasion, intravasation, and extravasation. The metastatic cascade is strongly dependent on factors like (1) loss of cell–cell adhesion capacity to allow malignant tumor cells to dissociate from the primary tumor mass, which depends on change in tumor cell plasticity by morphological and phenotypical conversions via the process of epithelial to mesenchymal transition (*EMT*); (2) changes in cell–matrix interaction, via degradation of the extracellular matrix (ECM) by a series of tumor-associated proteases, such as serine proteases, cysteine proteases, and metalloproteinases (MMPs) and (3) angiogenesis, which provides the means of supply of nutrients to and removal of waste products from the tumor site as well as provides a route for the detached tumor cells to enter the circulatory system and metastasize to distant sites [113,114,115].

The ability of lung cancer cells to resist or even thrive under hypoxia is a potent tumor microenvironmental factor that influences the behavior of tumors and stromal cells to promote metastasis and aggressiveness of lung cancer cells [111,112,116]. At the molecular level, hypoxia results in the stabilization of hypoxia inducible factor (HIF)-1 and HIF-2 and overexpresses HIF-1α. Activation of transcription factor HIF-1α results in the expression of the vascular endothelial growth factor (VEGF), which promotes endothelial proliferation, migration, and vessel sprouting by endothelial cells, which results in angiogenesis, metastasis, and growth of tumor mass [117]. Thus, overexpression of HIF-1α has a strong correlation with high metastatic risk, poor prognosis, pathological types and grade, and the survival of patients with lung cancer [118]. Contrary to VEGF, pigment epithelium-derived factor (PEDF), a potent antiangiogenic molecule, counteracts the effect of VEGF to prevent neovascularization. Thus, the ratio of VEGF/PEDF is crucial for determining angiogenesis and tumor growth [119]. Thus, to understand the Berberine-induced antiangiogenic effects, Fu et al. demonstrated the potency of Berberine in preventing HIF-1α expression in A549 lung cancer cells, which thereby inhibited the HIF-1α/VEGF signaling axis, resulting in the reduction of the VEGF/PEDF ratio in lung cancer cells [89].

Peng et al. demonstrated the potential of Berberine in significantly reducing the motility and invasion ability of highly metastatic A549 cells at non-cytotoxic concentrations. Berberine treatment suppressed the mRNA expression of extracellular matrix–degrading proteinases MMP2 and urokinase-plasminogen activator (u-PA), which are closely associated with the invasive and metastatic potential of lung cancer cells [93,120]. Further, the expression of tissue inhibitor of metalloproteinase (TIMP-2), a specific endogenous inhibitor of MMP2, was shown to be enhanced with Berberine administration. The proposed mechanism of the antimetastatic potential of Berberine is due to the suppression of NF-κB, c-Fos, and c-Jun protein expression as well as the inhibition of DNA binding activity of NF-κB and AP-1, involved in transcription regulation of MMPs or u-PA secretion [93]. Another study carried out in a Lewis lung carcinoma model showed that at lower drug dose, Berberine failed to affect the growth of tumors at the implanted site but significantly inhibited spontaneous mediastinal lymph node metastasis by lung cancer cells into the lung parenchyma. The antimetastatic effect was proposed due to the Berberine-mediated inhibition of AP-1 transcriptional activity, which subsequently reduces the expression and secretion of u-PA [121]. Epigenetic regulation of Berberine-induced downregulation of HDAC expression and activity, indicated by a reduction in MMP-2 and MMP-9 mRNA and protein levels, also demonstrated suppression of the invasive and metastatic potential of A549 lung cancer cells [73]. Other than the direct antimetastatic potential on lung metastasis, Berberine treatment can significantly reduce the lung metastasis from hepatocellular carcinoma by downregulating the expression of Inhibitor of DNA Binding 1 (Id-1) at the transcriptional level by inhibiting the Id-1 promoter activity [122]. Similarly, Berberine inhibited pulmonary metastatic tumor nodule formation in a B16F-10 melanoma tumor mice model by downregulating the expression of prometastatic genes such as *MMPs*, *ERK 1/2*, prolyl hydroxylase, and lysyl oxidase and upregulating the expression of the metastasis suppressor gene *Nm23* [95].

Zheng et al. showed the inhibitory effect of Berberine in suppression of epithelial-mesenchymal transition (*EMT*), which plays a crucial role in enhancing invasiveness, metastasis, resistance to treatment, and development of cells with stem cell-like characteristics in cancer. Mechanistically, Berberine treatment in lung cancer cell lines (i) inhibited the expression of HOX antisense intergenic RNA (HOTAIR), a long non-coding RNA (lncRNA), which shows a strong correlation with metastasis, drug resistance, and shortened overall survival of lung cancer patients and (ii) enhanced the expression of miR-34a-5p, a tumor-suppressive miRNA that suppresses the expression of several oncogenes, such as *KRAS* and *c-MYC*, to prevent and reverse tumorigenesis. The miR-34a-5p targets and inhibits zinc finger protein SNAI1 (snail) expression, a strong transcriptional repressor of the *E-cadherin*. Thus, overall suppression of EMT is mediated by Berberine-induced alteration in the interaction between HOTAIR and miR-34a-5p, resulting in induction of *E-cadherin* and reduction of vimentin and snail expression [123]. While another study showed that inhibition of *EMT* in in vitro and in vivo A549 cell models by Berberine is mediated by inhibition of the effects of TGF-β 1 on *EMT* and suppression of the expression of *EMT*-inducing transcription factors, Snail1 and Slug [94].

## 5. Berberine-Based Combination Therapy

Other than eliciting direct anticancer effects, randomized trials in NSCLC patients treated with radiotherapy showed that Berberine treatment with a dose of 20 mg/kg once a day for 6 weeks significantly reduced the incidence of radiation-induced lung injury, with improved basal pulmonary function. Furthermore, the treatment strategy resulted in the reduction of the expression of soluble intercellular adhesion molecular-1 (sICAM-1) and transforming growth factor-beta-1 (TGF-β1), involved in the pathogenesis of radiation-induced inflammation [124]. Further, Berberine has been shown to overcome Doxorubicin (DOX)-mediated resistance and enhanced sensitivity of lung cancer cells to DOX. Molecular mechanistic results showed that Berberine can potentially inhibit the DOX-stimulated activation of *STAT3*, thus inhibiting cell proliferation and apoptosis induction in DOX resistant cells [99]. Additionally, inhibition of *MET* by Berberine functions to synergize its effects with an EGFR tyrosine kinase inhibitor Osimertinib, to overcome the acquired resistance in NSCLC cells to Osimertinib by induction of Bim-dependent apoptosis [125]. Certain reports have shown the potential of the synergistic responses of Berberine with the combination of melatonin, metformin, cinnamaldehyde, icotinib, and gefitinib to achieve enhanced anticancer effects by controlling several cancer crucial genes and signaling pathways involved in lung cancer cell growth, invasion, and metastasis [101,123,126,127,128]. In addition to the chemo-cytotoxic potential of Berberine, Peng et al. further showed the radio sensitizing potential of Berberine against both in vitro and in vivo NSCLC lung cancer models. Berberine-induced enhancement in ionizing radiation (IR)-mediated cytotoxicity against radioresistant A549 cells was attributed to the induction of G2/M phase cell cycle arrest and autophagic cell death. Synergistic treatment of Berberine with an 8 Gy Xray dose in a Lewis lung carcinoma mice model showed almost 48% and 22% reduction in tumor volume at 1.0 and 2.0 mg/kg of Berberine administration, respectively. Accordingly, this study explored the potential of Berberine for use as adjuvant therapy to treat lung cancer [129]. In addition, a single hybrid nanocarrier composed of Berberine and Zinc Oxide (ZnO) nanoparticles was shown to induce improved antiproliferation efficacy against A549 cells based on its chemo-photothermal therapeutic efficacy [130]. Thus, as a strategy for lung cancer–associated adjuvant therapy, a combination of conventional therapy or drugs with the potential phytochemical Berberine may improve the treatment outcome in lung cancer patients

## 6. Limitations and Future Perspective

Despite having anticancer potential, Berberine suffers from several different limitations, which restrict its clinical applications. For example, a study showed the ineffectiveness of Berberine in killing lung cancer stem cells, demonstrated by an increase in the survival of the side population fraction of H460 lung cancer cells, which represents cancer stem cells, upon Berberine treatment alone or in combination with 5-Flurouracil (5-FU) [97]. Such findings warrant further investigations for the cautious application of Berberine in the treatment of cancers. One of the major disadvantages is the poor pharmacokinetics of Berberine due to its poor absorption rate, which results in its quick elimination from the body. Thus, high doses of Berberine are needed to achieve the desired therapeutic effects. Higher Berberine concentration is also unattainable under human physiological conditions, considering the less effective concentration at the tumor site. Numerous studies have attempted to resolve this limitation through the alteration of Berberine’s structure as well as synergistic administration of Berberine with a nano-based delivery system and/or an absorption enhancer [131,132,133]. For instance, dihydro Berberine, a derivative of Berberine that has been structurally modified, improves the absorption rate. However, Berberine can be reduced to dihydro Berberine in the intestine via enzymatic controlled reactions performed by the bacterial nitroreductase present in the gut microbiota. Invariably, this Berberine derivative can equally be reversed (through the oxidative process) back to Berberine via certain reactions in the intestine requiring no enzymes [134,135]. Hence, concomitant administration of probiotics could alleviate Berberine’s bioavailability. Conversely, Berberine’s oral bioavailability can be improved by making use of absorption enhancers like sodium caprate, which exerts its effect by increasing its cellular permeability [136]. In addition, varieties of nanoparticle carriers have been discovered and used to enhance solubility and Berberine’s bioavailability. For example, nanostructured lipid carriers (NLCs) [137,138], micelles [139], and other lipid-based nanoparticles were shown to enhance oral bioavailability, solubility, and therapeutic outcome via Berberine’s encapsulation. Similarly, dendrimers [140], nanogels [141], chitosan [142], and all other polymeric-based nanoparticle carriers were also shown to improve Berberine’s pharmacokinetics. Further, as discussed above, combining different anticancer therapies offers the advantages of using lower drug doses along with a better therapeutic outcome [52]. Other than the above-stated treatment options, utilizing the strong absorption in the blue wavelength region, Berberine can also be investigated as a potent chemo-photosensitizing agent against localized lung tumor mass. Photodynamic therapy, an adjuvant therapy, offers several advantages, including non-invasiveness and localized treatment with less systemic toxicity [143].

## 7. Conclusions

**Figure 5:**
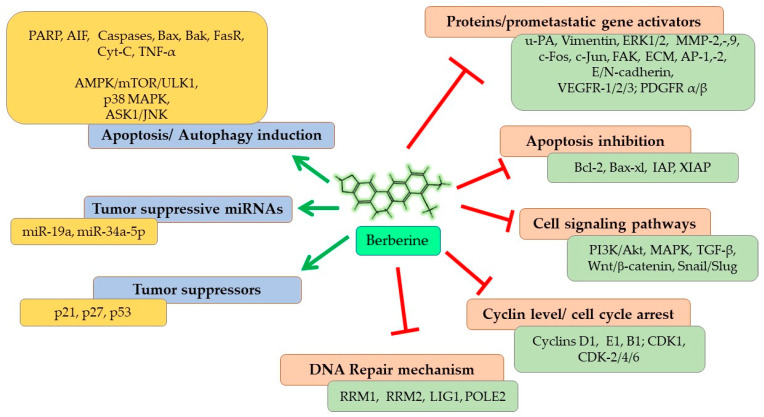
An overall summary of the molecular effects of Berberine resulting in antineoplastic and antimetastatic efficacy against lung cancer. Arrows show induction; lines without arrows show inhibition.

In this review, we aimed to discuss the molecular mechanism of the antineoplastic and antimetastatic potentials of Berberine, highlighting some of the principal signaling pathways, summarized in Figure 5. Therefore, Berberine is a potential candidate as a relatively safe and promising adjuvant therapy to cure cancers, especially the highly prevalent lung cancer, alone and in combination with several different conventional and recent cancer therapies.

## Figures and Tables

**Table 1:** Comparison of therapeutic potential and induced mechanisms of various Berberine derivatives against in vitro lung cancer models.

Berberine Derivatives	Therapeutic Potency Compared to Berberine	Proposed Induced Mechanism of Cell Death
9-O position modified BBR derivative with long alkyl chain branched by hydroxyl and methoxycarbonyl group [78]	3.6-fold higher intracellular concentration and 60-fold higher anti-proliferation activity than Berberine	Selectively accumulate in mitochondria, resulting into drastic increase in mitochondrial functional dysregulation, by decreasing oxygen consumption rate and mitochondrial membrane potential along with increased mitochondrial fragmentation, thus inducing apoptosis in A549 cells.
9-O-butylberberrubine bromide, 9-O-hexylberberrubine bromide, 9-O-octylberberrubine bromide, 9-O-decylberberrubine bromide, and 9-O-dodecylberberrubine bromide [10]	3 to 50 times lower IC_50_ compared to Berberine	Localizes in mitochondria, induces cell cycle arrest at the G0/G1 phase, inhibits in vitro tumorigenesis and enhances autophagy without induction of apoptosis in A549 cells.
8-Cetylberberine [79]	10 times lower IC_50_ compared to BBR	Downregulates the PI3K- Akt pathway along with an upregulating p21 and p27 protein expression resulting into induction of both intrinsic apoptotic pathway and extrinsic apoptotic pathway, and G0/G1 phase cell cycle arrest in A549 cells.
9-O-isoprenylberberrubine bromide, 9-O-gernylberberrubine bromide, and 9-O-farnesylberberrubine bromide [77]	IC_50_ range from 0.6–12.9 μM, (IC_50_ for Berberine not determined)	Induces G0/G1 phase cell-cycle arrest, partial cellular apoptosis and autophagic flux blocking alongside suppression of in vitro tumorigenesis and tumor migration in A549, H23, and H1435 cells.

**Table 2:** Summary of antiproliferative and antimetastatic potential of Berberine on several reported in vitro and in vivo lung cancer models.

Antiproliferative Property	
**In Vitro** **Cell lines**	**Effect and Mechanism**
A549 and PC9 cells	Cellular apoptotic induction through activation of the ROS/ASK1/JNK and p38 MAPK signaling pathway Inhibition of cell proliferation via MMP-2, Bcl-2/Bax, and Jak2/VEGF/NF-Kb/AP-1 signaling pathways [86,87]
H1299 and A549 H460	Repress cell proliferation through proliferative kinase signaling inhibition [88] Induction of cell cycle arrest at G0/G1 [89]
A549, PC9, H460, Beas-2b, H1299, 293T cells NCI-H460, A549, and NCI –H1299 cells	Induction of cellular apoptosis via miR19a/TF/MAPK signaling pathway [90] Berberine-induced DNA damage resulted in apoptosis, whereby Berberine accelerated the degradation of SW1-independent—3 transcription regulator family member A (Sin3A) protein and down-regulation of protein level of TOP2 β [91]
**In vivo** **models**	**Effect and Mechanism**
C57BL/6 mice BALB/c nude mice	Triggers enlargement of tumor necrosis area [92] Represses tumor growth through Sin3A/TOP2 β pathway [91]
Athymic nude mice nu/nu nude mice	Prevents tumor growth [88] Initiates cell cycle arrest at G1 phase through Akt/CREB signaling pathway [86]
**Anti-Metastatic Property**	
**In vitro** **Cell lines**	**Effect and Mechanism**
A549 cells	Prevents/inhibits cell proliferation/invasion via *MMP-2, -9* disruption, and *u-PA* downregulation [93]
A549 cells	Berberine inhibits HIF-/α/VEGF signaling, thereby reducing VEGF/PEDF ratio [89]
A549 cells	Berberine inhibits TGF-β1 effects on *EMT* and suppresses the expression of EMT inducing transcription factors Snail and Slug [94] (Qi et al., 2014)
**In vivo** **models**	**Effect and Mechanism**
B160F-10 tumor mice model	Berberine downregulates expression of pro-metastatic genes such as *MMPs, ERK 1/2*, /Proly/hydroxylase [95]
A549 cells	Berberine inhibits TGF-β1 effects on *EMT* and suppresses the expression of *EMT* inducing transcription factors Snail and Slug [94]
